# Cardiovascular Outcomes after Paclitaxel-Coated Balloon Angioplasty versus Drug-Eluting Stent Placement for Acute Coronary Syndrome: A Systematic Review and Meta-Analysis

**DOI:** 10.3390/jcm13051481

**Published:** 2024-03-04

**Authors:** Yuki Kondo, Tetsuya Ishikawa, Masatoshi Shimura, Kota Yamada, Tomoaki Ukaji, Yohei Tamura, Miona Arai, Kahoko Mori, Taro Takeyama, Yuichi Hori, Itaru Hisauchi, Shiro Nakahara, Yuji Itabashi, Sayuki Kobayashi, Isao Taguchi

**Affiliations:** Department of Cardiology, Dokkyo Medical University Saitama Medical Center, 2-1-50 Minamikoshigaya, Koshigaya 343-8555, Saitama, Japan; k-yuki@dokkyomed.ac.jp (Y.K.); m-simur@dokkyomed.ac.jp (M.S.); yamanyaman219@yahoo.co.jp (K.Y.); tomoaki_ukaji@yahoo.co.jp (T.U.); y-tamura828@dokkyomed.ac.jp (Y.T.); m-arai536@dokkyomed.ac.jp (M.A.); mo11thca@yahoo.co.jp (K.M.); t-taro@dokkyomed.ac.jp (T.T.); hhkxn763@yahoo.co.jp (Y.H.); hisauchi@dokkyomed.ac.jp (I.H.); nshiro@dokkyomed.ac.jp (S.N.); y-itabashi@dokkyomed.ac.jp (Y.I.); sayuki@dokkyomed.ac.jp (S.K.); billytaguchi@gmail.com (I.T.)

**Keywords:** coronary drug-coated balloon, myocardial infarction, primary stenting

## Abstract

**Background**: We conducted a systematic review and meta-analysis to examine the feasibility of paclitaxel-coated balloon (PCB) angioplasty for de novo lesions in patients with acute coronary syndrome (ACS) by comparing with drug-eluting stent (DES) placement. **Methods**: By a systematic literature search, nine (five randomized controlled, two retrospective propensity-score matched, and two retrospective baseline-balanced) studies comparing the midterm clinical and angiographic outcomes after PCB angioplasty and DES placement were included, yielding 974 and 1130 ACS cases in PCB and DES groups, respectively. Major adverse cardiac event (MACE) was defined as a composite of cardiac mortality (CM), all-cause mortality (ACM), myocardial infarction (MI), target vessel revascularization (TVR), and target lesion revascularization (TLR). Late luminal loss (LLL) and bleeding events (BLD) were also estimated. **Results**: The frequencies of MACE in PCB and DES groups were 8.42% and 10.62%, respectively. PCB angioplasty had no significant impacts on all of MACE (risk ratio: 0.90, 95%CI: 0.68–1.18, *p* = 0.44), CM, ACM, MI, TVR, TLR, BLD, and LLL, compared to DES placement in random-effects model. **Conclusions**: The present systematic review and meta-analysis showed the feasibility of PCB angioplasty for the de novo lesions in patients with ACS in comparison with DES placement by the emergent procedures.

## 1. Introduction

Drug-coated balloon (DCB) angioplasty for coronary in-stent restenosis (ISR) inside drug-eluting stent (DES) and for de novo lesions in small coronary vessels in patients with chronic coronary syndrome (CCS) by the elective procedures has been feasible in comparison with DES [1,2,3]. In the present DES era, since there still remained the unresolved concerns of DES, such as ISR and devastating stent thrombosis, DCB angioplasty has required further evidences to extend the clinical indications beyond ISR inside DES and small vessels [1,2,3]. Of them, on the basis of the growing evidences of DCB angioplasty for CCS, de novo lesions in large coronary vessels, bifurcation without jailing the branches by DES, lesions in diabetic patients, and acute coronary syndrome (ACS) were considered to be the candidates of DCB angioplasty as an alternative of DES [1,2,3].

The most up-to-date 2023 European Society of Cardiology (ESC) Guideline for the management of ACS [4] and the major recent reviews focusing on DCB angioplasty [1,2,3] stated the possibility of DCB angioplasty as the efficacious method of “stent-less PCI” and “leave-nothing-behind strategy”, in the reperfusion therapy for ACS. In the guideline [4] and statements [1,2,3], a non-inferiority of DCB angioplasty compared to DES placement for the assigned ACS was mentioned by referring a few reports [5,6] without showing the evidence level. At present, there has been no systematic review or meta-analysis comparing the mid-term cardiovascular outcomes between DCB angioplasty and DES placements for ACS by including all of cases of STEMI, non-STEMI (NSTEMI), and unstable angina (UA).

In the present study, we conducted a systematic review and a meta-analysis to examine the feasibility of DCB angioplasty for de novo lesions in patients with ACS by comparing the mid-term cardiovascular outcomes with those of recent advanced DES. For this purpose, the randomized clinical trials and retrospective studies comparing the outcomes of DCB angioplasty with those of DES placement under the well-balanced or propensity-score matched baselines were screened. Of several DCBs, only paclitaxel-coated balloon (PCB) including SeQuent Please (B. Braun, Melsungen, Germany) was enrolled to ensure the effects of DCB. Therefore, the eligible nine studies comparing the several clinical and angiographic outcomes between PCB angioplasty and DES placement for de novo coronary lesions in patients with ACS with more than six months clinical observational intervals were included as the first meta-analysis showing the feasibility of PCB angioplasty for ACS.

## 2. Materials and Methods

### 2.1. Systematic Review Process

This meta-analysis was performed according to the Preferred Reporting Items for Systematic Reviews and Meta-analyses (PRISMA) guidelines [7]. The PRISMA 2020 checklist is attached in the table of Supplement (Table S1). This systematic review was not registered. Ethics approval was not required for this meta-analysis because none of the patients at our institute were included.

We conducted searches for published studies indexed in MEDLINE (PubMed) and Cochrane library in September 2023 and January 2024 using the terms “drug coated balloon coronary”, “drug coated balloons”, “acute coronary syndrome”, “acute myocardial infarction”, “ST elevation myocardial infarction”, “non-ST elevation myocardial infarction”, and “unstable angina”. The article types were either of “clinical trial” or “randomized controlled trial”.

The PRISMA flow diagram of the selection process: A total of 2025 potentially relevant articles in PubMed and Cochrane library were identified by Y.K. and T.I., respectively. The selection process included the following: identification, screening, eligibility, and inclusion of full-text articles published after 2016 [8] in English.

The eligibility criteria were as follows:

(1) Clinical diagnosis of acute coronary syndrome (ACS): as a collective term of ACS, or including independently ST-elevated myocardial infarction (STEMI), or non-ST-elevated myocardial infarction (NSTEMI), or unstable angina (UA).
(2) Lesion characteristics: de novo culprit lesions of ACS in native coronary artery.
(3) Methods to adjust the patient baselines: randomized controlled trial (RCT), or retrospective studies using a propensity-score matched analysis, or baseline balanced studies.
(4) Final device of emergent PCI: paclitaxel-coated balloon (PCB) including SeQuent Please (B. Braun, Melsungen, Germany) as the PCB group. Any second or third-generation DESs were exclusively placed as the DES group including bailout DES placement after major coronary dissections during the PCB angioplasty procedure.
(5) Estimated clinical outcomes: major adverse cardiac event (MACE) during more than 6 months observational interval consisted of either cardiac mortality (CM), or all-cause mortality (ACM), myocardial infarction (MI), or target vessel revascularization (TVR), or target lesion revascularization (TLR). The other estimate was major bleeding events (BLD) estimated according to BARC definition mainly 3b and 5 (Study No. 1 estimated BARC from 1 to 5). In addition, the mean magnitude of late luminal loss (LLL) was also estimated.

**Figure 1:**
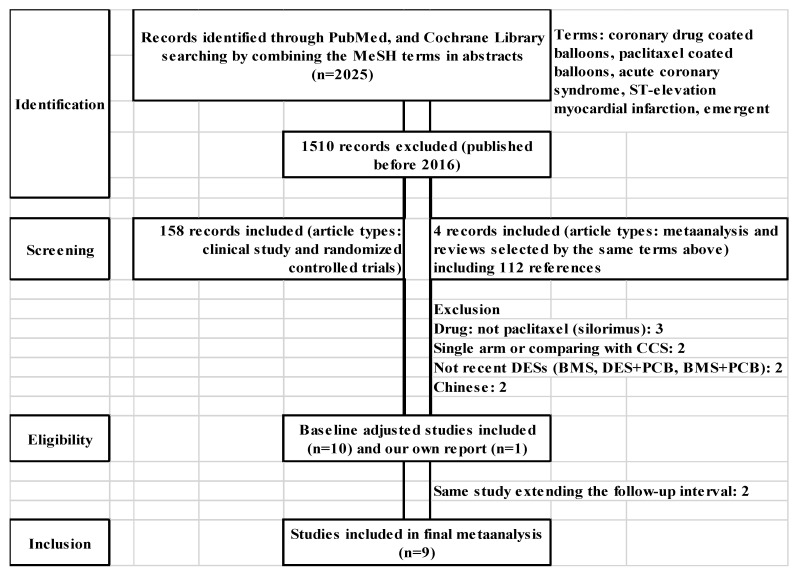
Flow chart of the systematic review. Among the 2025 studies, 9 studies were included. CCS: chronic coronary syndrome, BMS: bare-metal stent, DES: drug-eluting stent, PCB: paclitaxel-coated balloon.

Finally, there were 9 eligible studies estimating, yielding 974 cases of PCB angioplasty and 1130 cases of DES placement (Figure 1).

Assessment of the included studies and meta-analysis.

Data on the following ACS-related factors were collected from the 9 studies. 

Study design, baselines, and procedural characteristics of 9 studies (Table 1). Study number (No. 1–9), author, (the name of clinical study), and the Journal, study design, type of included ACS and population, definition of major adverse cardiac events (MACE), and mean follow-up interval. Inclusion and exclusion criteria are summarized in Table S2.

Baselines of patient, angiographic, lesion characteristics of 9 studies (Table 2): Mean age, percentages of males, diabetes, smokers, Killip classification of I and IV, culprit of left descending artery (LAD), thrombus-containing lesion or conducting aspiration (pre-aspiration), and calcified lesion in PCB and DES groups are shown, respectively. 

Characteristics of final device and dual anti-platelet therapy (DAPT) of 9 studies (Table 3): used PCB, used DES, percentages of bailout stent implantation, mean balloon diameters of PCB and DES (mm) or the explanation about the balloon size, mean total balloon lengths of PCB and DES (mm), and regimen and duration of DAPT are summarized.

Angiographic parameters: baseline reference vessel diameter (mm), minimal lumen diameter and percent diameter stenosis after PCI, mean late luminal loss (LLL) (mm) in the PCB and DES groups with *p*-values, and percentage of late lumen enlargement (LLE: minus LLL) are summarized in Table 4.

Lesion preparation for the culprit lesion, treatment of dissection during PCB angioplasty, and thrombectomy are summarized (Table S3).

### 2.2. Statistics

**Figure 2:**
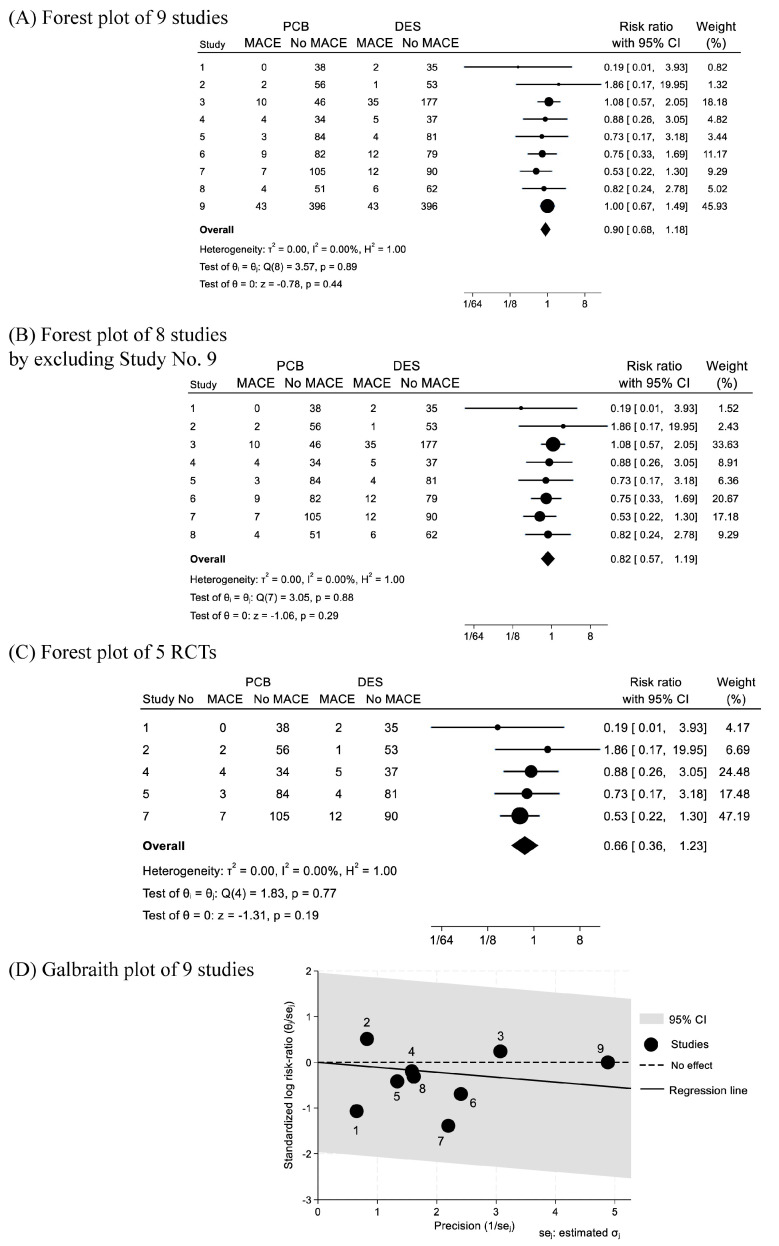
Forest plots of (**A**) all of 9 studies, of (**B**) 8 studies excluding Study No. 9, and of (**C**) 5 RCTs, and (**D**) Galbraith plot of all of 9 studies for comparison of major adverse cardiac events (MACE) between paclitaxel-coated balloon (PCB) angioplasty and drug-eluting stent (DES) placement in patients with acute coronary syndrome. Definitions of MACE of 9 studies were summarized in Table 1. In the Forest plots, the black circles represent the point estimate, and the size of the circle is proportional to the weight given to each study in the meta-analysis. Horizontal solid black lines represent 95% confidence intervals (CI). The bottom diamond represents the summary estimate (size of the diamond = 95%CI). As the sensitivity analyses of the main estimate of the present study (**A**), two Forest plots, defining in the text, were shown. In Galbraith plot (**D**), regression line (solid line) with the area of 95%CI (grey zone) was shown. The black circles represent the studies, and the numbers corresponded to the included study number. All of the studies (1–9) were within the range of 95%CI.

**Figure 4:**
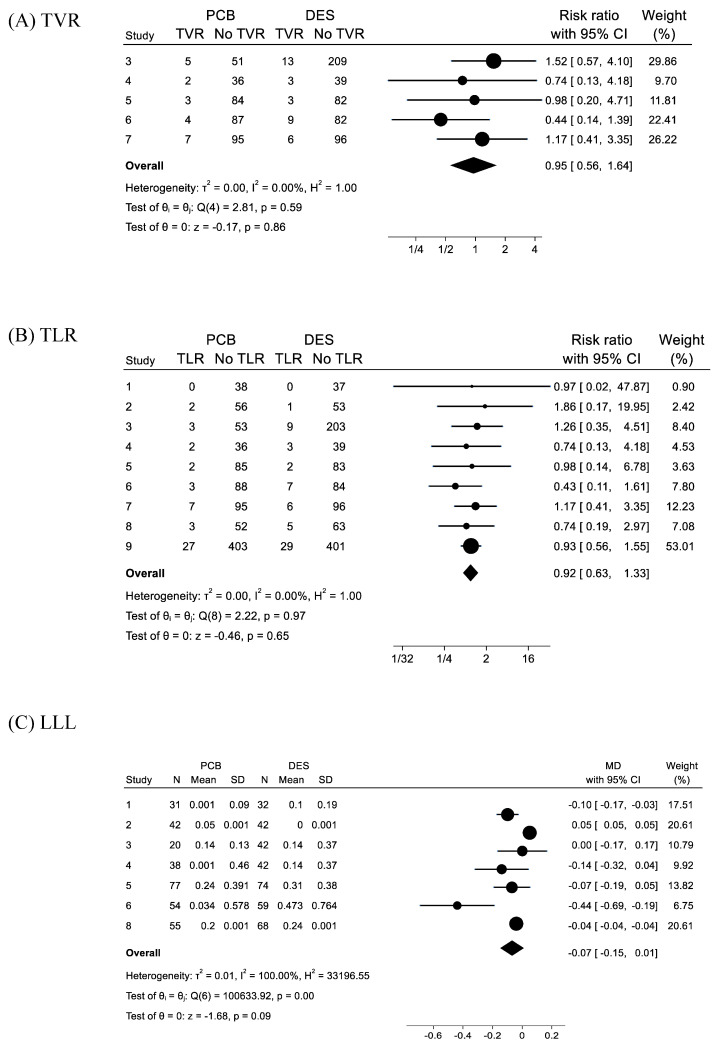
Forest plots for comparisons of (**A**) target vessel revascularization (TVR), (**B**) target lesion revascularization (TLR), and (**C**) late luminal loss (LLL) between paclitaxel-coated balloon (PCB) angioplasty and drug-eluting stent (DES) placement in patients with acute coronary syndrome. The explanations of Forest plots were same with Figure 2. In (**C**), the values of N (number of cohort), Mean (mean magnitude of LLL), and SD (standard deviation) of the PCB and DES groups of 7 studies, and mean differences (MD) with 95%CI of 7 studies were shown. The values of mean LLL and SD referred from Table 4 and modified as mentioned in the text.

The random-effects model was used to calculate risk ratios (RRs) and 95% confidence intervals (CIs), and heterogeneity (*I*^2^) analysis was performed using STATA version 18 (Lightstone corp., Tokyo, Japan), using the commands of “meta esize”, “meta sum”, and “meta forestplot, eform” for the categorical data of MACE, CM, ACM, MI, TVR, TLR, and BLD. Sensitivity analyses of the key estimate of MACE by including all of the 9 studies (Figure 2A), forest plots of MACE (1) by excluding the Study No. 9 whose weight was the highest and as high as approximately 45% (Figure 2B), and (2) by only including 5 RCTs (Figure 2C) were performed. In addition, the heterogeneity of the forest plot of the key estimate of MACE was very low (Figure 2A). Therefore, in order to further estimate the precision of the included 9 studies, Galbraith plot for the comparison of MACE in PCB angioplasty and DES placement, using the commands of “meta galbraithplot”, is shown in Figure 2D. Individual precisions are shown by assigning the study numbers in Figure 2D. Since LLL were continuous data, mean difference (MD) with the 95%CI was calculated as the effect size, using the commands of “meta esize”, “meta sum”, and “mean forestplot”. In this analysis of LLL, minus value of LLL and not-available standard deviation were treated as 0.001, in order to implement the analysis (Figure 4C). As a sub-analysis of the present study for ACS, the 4 studies including only STEMI were selected, and forest plots for the comparisons of MACE, CM, ACM, and TLR between PCB angioplasty and DES placement were estimated. *I*^2^ statistic was used to assess the heterogeneity between studies with values 0–30%, more than 30–60%, and more than 60% corresponding to low, moderate, and high degree of heterogeneity, respectively. 

## 3. Results

### 3.1. A Flow of a Systematic Review

The PRISMA flow diagram of the selection process is shown in Figure 1. As mentioned above in Materials and Methods, nine studies were eligible to be included. the studies were assigned the study numbers according to the publication date: No. 1: Gobić D, et al. [9], No. 2: Vos NS, et al. [6], No. 3: Tan Q, et al. [10], No. 4: Hao X, et al. [11], No. 5: Wang Z, et al. [12], No. 6: Mizutani, et al. [13], No. 7: Mangner N, et al. [14], No. 8: Zhang YB, et al. [15], No. 9: Merinopoulos I, et al. [16].

### 3.2. Introductions of Nine Included Studies

The study design, inclusion and exclusion criteria, and definition of major adverse cardiac event (MACE) of the nine studies are summarized in Table 1 and Table S2. There were five randomized control studies (RCTs), and four retrospective studies. Of the four retrospective studies, two studies were applied with propensity-score matched analysis (PSM), and the baselines of two studies were well balanced.

The baselines of patient, angiographic, and lesion characteristics of nine studies are summarized in Table 2. In four studies, the cases of cardiogenic shock (Killip classification of 4) were excluded (Table 2 and Table S2). In one study, thrombus containing lesion was excluded. In four studies, severely calcified lesions were excluded (Table 2). 

The characteristics of used final devices and dual anti-platelet therapy (DAPT) are summarized in Table 3. There were five kinds of PCB. In three studies, the percentages of bailout stenting during PCB angioplasty procedure were mentioned with approximately 5%. All of the mean total balloon lengths were approximately 20 mm and nearly the same. In five studies, the mean duration of DAPT in the PCB group was shorter than in the DES group.

The methods of lesion preparation for the culprit lesion including thrombectomy, and the treatment of dissection during PCB angioplasty are summarized in Table S3. Usually, a semi-compliant balloon and scoring balloon were used to modify the culprit lesion. In the case of thrombus containing lesions, aspiration to reduce the thrombus burden was conducted. When the dissection types from C to F occurred, stents were placed to bailout from the PCB angioplasty procedure.

Angiographic parameters are summarized in Table 4. In five studies, the mean magnitudes of LLL in the PCB group were very small, less than 0.10 mm, and significantly smaller than those in the DES group. In two studies (No. 6 and 8), the percentages of late lumen enlargement (LLE) were reported to be approximately 50%, ranging from 48 to 56%.

#### 3.2.1. Major Adverse Cardiac Events (MACE) 

The definitions of MACE of nine studies are summarized in Table 1. In Figure 2A, the number of patients with (MACE) and without MACE (No MACE), and the total number of cohorts in the PCB and DES groups are shown. The statistical differences (*p*-values) were also referred from the reports. The frequencies of MACE in the PCB and DES groups were 8.42% (82/974) and 10.62% (120/1130), respectively. The risk ratio (RR) of PCB angioplasty for MACE was 0.90 with 95%CI of 0.68–1.19 (*p* = 0.44), and *I*^2^ = 0% (*p* = 0.89) (Figure 2A). In Figure 2B, a forest plot estimated MACE by excluding Study No. 9 is shown. This figure shows a sensitivity analysis of the main estimate (Figure 2A) by excluding the study of the largest weight. The RR of PCB angioplasty for MACE was 0.82 with 95%CI of 0.57–1.19 (*p* = 0.29), and *I*^2^ = 0% (*p* = 0.88) (Figure 2B). In addition, a forest plot estimated MACE by including five RCTs was shown in Figure 2C. The RR of PCB angioplasty for MACE was 0.66 with 95%CI of 0.36–1.23 (*p* = 0.19), and *I*^2^ = 0% (*p* = 0.77) (Figure 2C).

A Galbraith plot (Figure 2D) showed that all of the nine studies were within the range of 95%CI (grey zone), expressing the very low value of the heterogeneity. The downward to the right regression line corresponded to the RR value of 0.90 in the forest plot (Figure 2A). Since the value of RR of Study No. 9 was one, the black circle was on the dashed line (no effect). The values of precision of the study were increased from Study No. 1 (lowest), 2 (second lowest), 5, 4, 8, 7, 6, 3, to No. 9 (highest), in order. 

#### 3.2.2. Cardiac Mortality

**Figure 3:**
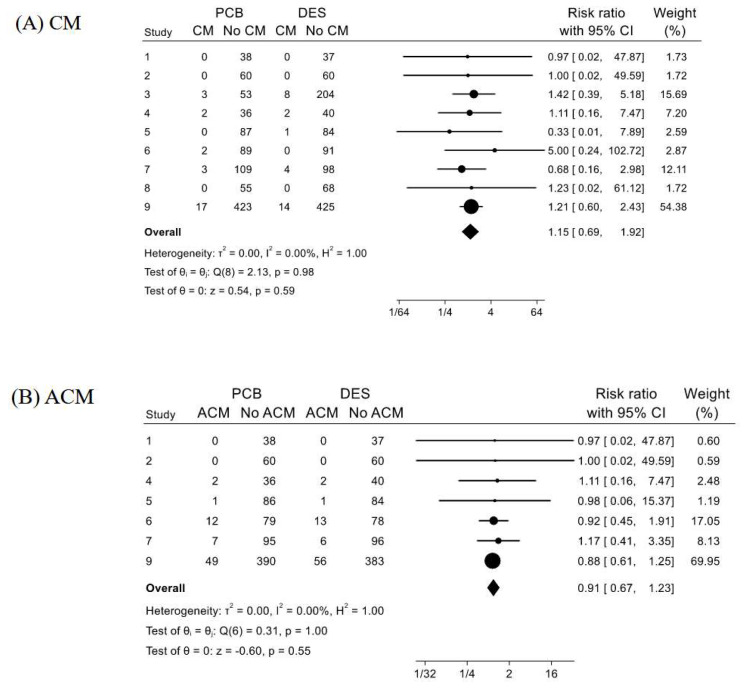

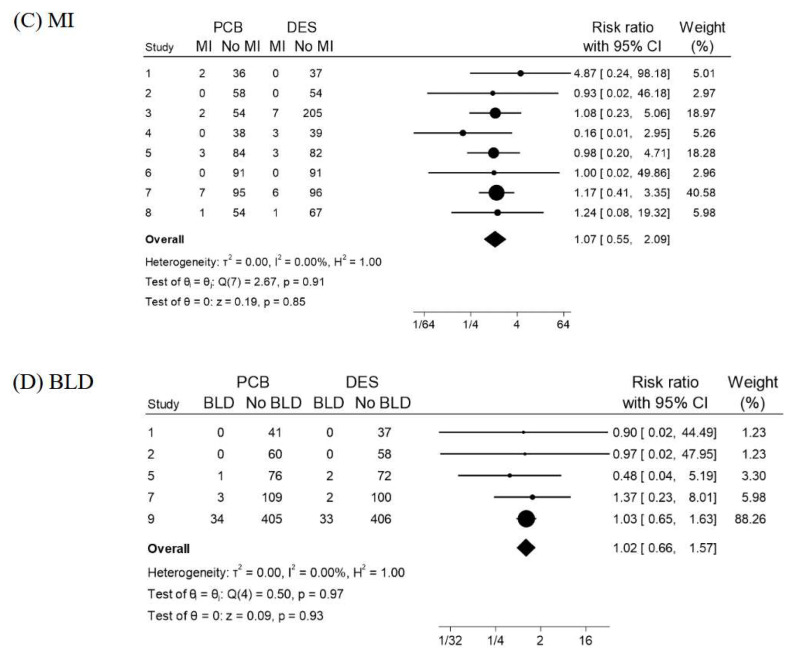
Forest plots for comparisons of (**A**) cardiac mortality (CM), (**B**) all-cause mortality (ACM), (**C**) myocardial infarction (MI), and (**D**) major bleeding events (BLD) between paclitaxel-coated balloon (PCB) angioplasty and drug-eluting stent (DES) placement in patients with acute coronary syndrome. The explanation about the Forest plots was the same with Figure 2.

The frequencies of cardiac mortalities (CM) in the PCB and DES groups composed by all the nine studies were 2.77% (27/976) and 2.55% (29/1136), respectively. In the forest plot (Figure 3A), the RR of PCB angioplasty for CM was 1.15 with 95%CI of 0.69–1.92 (*p* = 0.59), and *I*^2^ = 0% (*p* = 0.98).

#### 3.2.3. All-Cause Mortality

The frequencies of all-cause mortalities (ACM) in the PCB and DES groups composed of seven studies without two studies (No. 3 and 8) were 8.21% (71/865) and 9.11% (78/856), respectively. In the forest plot (Figure 3B), the RR of PCB angioplasty for ACM was 0.91 with 95%CI of 0.67–1.23 (*p* = 0.55), and *I*^2^ = 0% (*p* = 1).

#### 3.2.4. Myocardial Infarction

The frequencies of myocardial infarction (MI) in the PCB and DES groups composed by 8 studies without Study No. 9 were 2.80% (15/535) and 2.89% (20/691), respectively. In the forest plot (Figure 3C), the RR of PCB angioplasty for MI was 1.07 with 95%CI of 0.55–2.09 (*p* = 0.85), and *I*^2^ = 0% (*p* = 0.91).

#### 3.2.5. Major Bleeding Events (BLD)

The frequencies of major bleeding events (BLD) in the PCB and DES groups composed of five studies without studies No. 3, 4, 6, and 8 were 5.21% (38/729) and 5.21% (37/710), respectively. In the forest plot (Figure 3D), the RR of PCB angioplasty for TVR was 1.02 with 95%CI of 0.66–1.57 (*p* = 0.93), and *I*^2^ = 0% (*p* = 0.97).

#### 3.2.6. Target Vessel Revascularization (TVR)

The frequencies of target vessel revascularization (TVR) in the PCB and DES groups composed by five studies without studies No. 1, 2, 8, and 9 were 5.47% (21/384) and 6.39% (34/532), respectively. In the forest plot (Figure 4A), the RR of PCB angioplasty for TVR was 0.95 with 95%CI of 0.56–1.64 (*p* = 0.86), and *I*^2^ = 0% (*p* = 0.59).

#### 3.2.7. Target Lesion Revascularization (TLR)

The frequencies of target lesion revascularization (TLR) in the PCB and DES groups composed of all the nine studies were 5.08% (49/965) and 5.53% (62/1121), respectively. In the forest plot (Figure 4B), the RR of PCB angioplasty for TVR was 0.92 with 95%CI of 0.63–1.33 (*p* = 0.65), and *I*^2^ = 0% (*p* = 0.97).

#### 3.2.8. Late Luminal Loss (LLL)

The parameters of quantitative coronary angiography (QCA) including mean magnitude with standard deviation (SD) of LLL, and the *p*-values of comparison are summarized in Table 4. Of nine studies, the LLL data of seven studies were included. Of them, LLL of the PCB group in four studies were significantly smaller than those of the DES group. In two studies, mean LLLs were minus values (Studies No. 1 and 4). The percentages of LLE were referred from two reports. In the forest plot (Figure 4C), the MD (mean difference) of PCB angioplasty for the comparison of LLL with DES placement was −0.07 with 95%CI of −0.15–0.03 (*p* = 0.09), and *I*^2^ = 100% (*p* < 0.01). 

#### 3.2.9. Cardiovascular Outcomes in a Subset of STEMI

**Figure 5:**
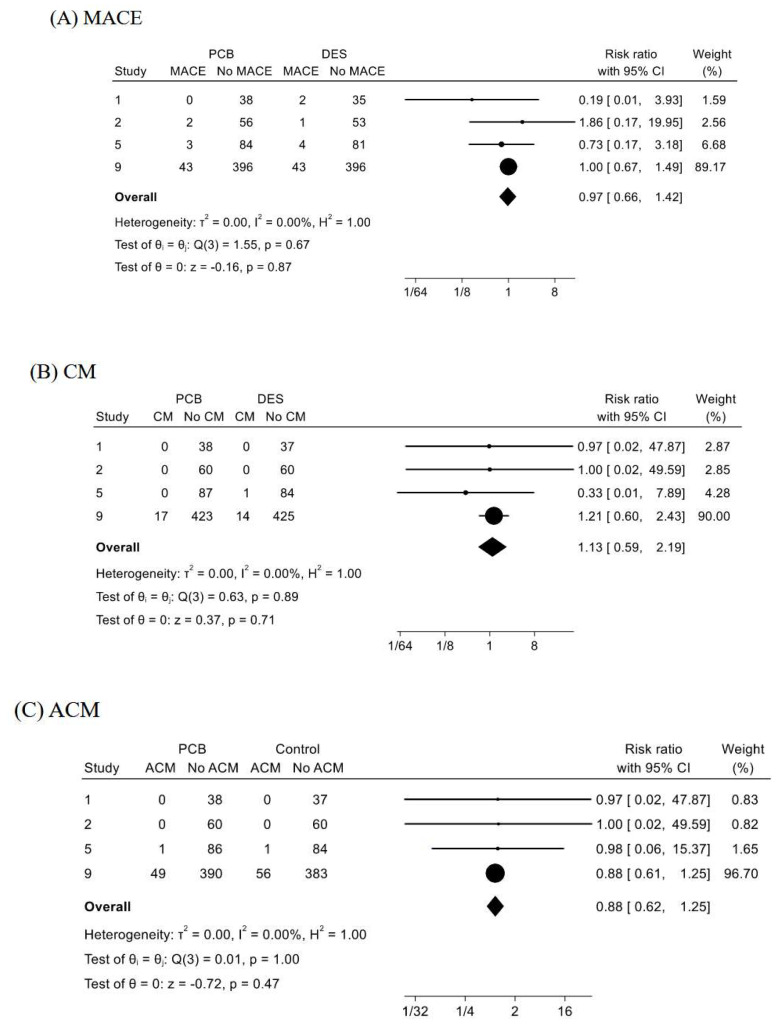

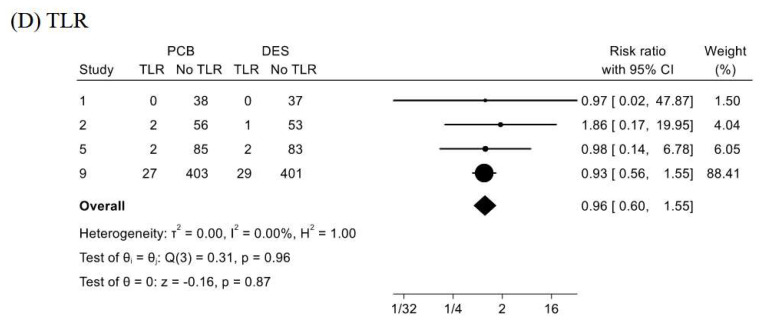
Forest plots for comparisons of (**A**) MACE, (**B**) cardiac mortality (CM), (**C**) all-cause mortality (ACM), and (**D**) target lesion revascularization (TLR) between paclitaxel-coated balloon (PCB) angioplasty and drug-eluting stent (DES) placement in patients with ST-elevated myocardial infarction (STEMI). As the sub-analysis, impacts of PCB angioplasty on the 4 cardiovascular outcomes in patients with STEMI were compared with those of DES placement. The explanations of Forest plots were same with Figure 2.

In Figure 5, forest plots for estimating (A) MACE, (B) CM, (C) ACM, and (D) TLR in patients with STEMI between PCB angioplasty and DES placement are shown as a sub-analysis of ACS. The frequencies of MACE, CM, ACM, and TLR composed of four studies in the PCB and DES groups were 7.72% (48/622) and 8.13% (50/615), 2.72% (17/624) and 2.42% (15/621), 8.01% (50/624) and 9.18% (57/621), and 5.06% (31/613) and 5.28% (32/606), respectively. In the forest plots, the RRs of PCB angioplasty for MACE was 0.97 with 95%CI of 0.66–1.42 (*p* = 0.87), and *I*^2^ = 0% (*p* = 0.67), for CM was 1.13 with 95%CI of 0.59–2.19 (*p* = 0.71), and *I*^2^ = 0% (*p* = 0.89), for ACM was 0.88 with 95%CI of 0.82–1.25 (*p* = 0.47), and *I*^2^ = 0% (*p* = 1.00), and for TLR was 0.96 with 95%CI of 0.60–1.55 (*p* = 0.87), and *I*^2^ = 0% (*p* = 0.96), respectively.

## 4. Discussion

The present systematic review and meta-analysis showed the feasibility of paclitaxel-coated balloon (PCB) angioplasty for patients with acute coronary syndrome (ACS) by comparing eight major cardiovascular outcomes with the recent drug-eluting stent (DES) placement. By including nine studies, yielding more than 2000 ACS cases, both the clinical safety and the angiographic efficacy of PCB angioplasty were statistically confirmed in all of the estimates of PCB angioplasty compared to DES placement, i.e., in the estimations of major cardiovascular outcomes (MACE) (Figure 2A) with two sensitivity analyses (Figure 2B,C), cardiac mortality (Figure 3A), all-cause mortality (Figure 3B), myocardial infarction (Figure 3C), bleeding events (Figure 3D), target vessel revascularization (Figure 4A), target lesion revascularization (Figure 4B), and late luminal loss (LLL) (Figure 4C). Therefore, to our knowledge, the present study is the first systematic review and meta-analysis showing the feasibility of PCB angioplasty for patients with ACS by the emergent procedures in comparison to the primary DES placement.

ACS has been considered to be one of the candidates of the clinical indication of PCB angioplasty as an alternative of primary DES placement on the basis of the evidences of PCB angioplasty for chronic coronary syndrome (CCS) [1,2,3,4]. The most up-to-date 2023 European Society of Cardiology (ESC) Guideline for the management of ACS [4] and the clinical expert consensus document of the Task Force of the Japanese Association of Cardiovascular Intervention, Therapeutics (CVIT) on PCB angioplasty [3] stated the possibility of PCB angioplasty for ACS by referring the Study No. 2 (REVELATION randomized trial) [6] and PEPCAD NSTEMI trial [5]. Therefore, meta-analyses showing the feasibility of PCB angioplasty for ACS by comparing with DES placement have not been referred in the guideline and statements. The present meta-analysis screened the recent growing evidences of PCB angioplasty for ACS including all of the emergent cases of STEMI, NSTEMI, and unstable angina (UA). All of the baselines of the included studies were statistically balanced (Table 1). The adjustment of baselines is essential in the comparison between PCB angioplasty and DES placement, because DES was preferred for use in the complex lesions and in patients with severe hemodynamics (Table 2 and Table S2). Patients with cardiogenic shock and lesions with heavily calcification tended to be excluded (Table 1, Table 2 and Table S2), although those were the consistent predictors of adverse outcomes in patients with ACS. PEPCAD NSTEMI trial [5] was excluded because DES was not exclusively used for revascularization. The Galbraith plot for MACE showed the order of the precision of the included studies for estimating the impact of PCB angioplasty on MACE in comparison with DES placement (Figure 2D). The Study No. 2 [6] was the second lowest (Figure 2D). Therefore, by including the recent studies from Study No. 3 to No. 9 onward No. 2 with higher precision studies, the present systematic review and meta-analysis showed a novel significance of PCB angioplasty in the field of revascularization methods of ACS.

PCB angioplasty, a brief and single application of paclitaxel on the culprit plaque after lesion preparation, would exert the following several points of benefits for the treatment of ACS in the clinical settings. First, the guideline-recommended primary DES placement for ACS [4] has several adverse impacts during a long-term interval, such as a gradual increase in the incidence of very late stent thrombosis (VLST) owing to the delayed persistent inflammation on the vessel wall and/or neo-atherosclerosis [17]. PCB angioplasty could avoid the unresolved and serious clinical courses due to DES failure of VLST and in-stent restenosis (ISR). Recently, there was an increase in the population of younger aged ACS including STEMI, and the benefits of PCB angioplasty with long-term optimal medical therapy without coronary stent implantation for younger patients with ACS was reported [18]. Second, PCB angioplasty showed good angiographic outcomes at chronic phase represented in small LLL (Table 4). The small (less than 0.10 mm) mean magnitudes of LLL after PCB angioplasty were consistent in the cases of CCS by the elective procedures regardless of vessel sizes [1,19,20,21,22]. A small LLL gave low incidences of TLR including TVR; those in turn, contributed to the low and statistically equivalent frequencies of composite MACE compared to DES (Figure 2, Figure 3 and Figure 4). In cases of CCS, a net small LLL was composed by approximately half of minus LLL [20,21,22,23,24], i.e., late lumen enlargement (LLE). We previously reported a potential mechanism for LLE in cases of CCS [21], with the following four factors: (I) balloon angioplasty enlarged the lumen and vessel areas (vessel enlargement); (II) the distribution of paclitaxel suppressed the plaque and induced its regression; (III) minor dissections formed at any step further facilitated the diffusion of paclitaxel into the plaques; and (IV) almost all minor dissections formed peri-operatively were sealed in the chronic phase without LLL. These serial steps for establishing LLE were consistent with the reports estimating the intravascular parameters of PCB angioplasty including the relation of dissections [23,24]. This unique angiographic phenomenon at chronic phase after PCB angioplasty, LLE, was also observed in cases of ACS [13,15,24,25,26] (Table 4). Similarly, LLE was observed in approximately half of the angiographic followed-up lesions after PCB angioplasty for ACS (Table 4) as with after for CCS [20,21,22,23]. Greater lumen enlargement in ACS compared to CCS with the significant increase in the vessel and lumen area, and significant decrease in the plaque area [24] was reported. Thus, stent-less PCB angioplasty consistently made approximately half the ratio of LLE in not only CCS, but also ACS. These efficacies of PCB angioplasty on the angiographic outcomes were quite different from the past plain old balloon angioplasty (POBA) conducted prior to the bare-metal stent (BMS) era. “Stent-Like” results with previous POBA strategy in primary PCI for ACS were not feasible, found by comparing with primary BMS placement [27]. In order to exert these advantages by final paclitaxel application in the culprit segment, the lesion preparation with smaller semi-compliant balloon and scoring balloon, and thrombus aspiration without significant dissection and without decrease in the Thrombolysis in myocardial Infarction (TIMI)-grade flow are very important in the reperfusion therapy for PCB angioplasty (Table S3). Particularly, in the PCB angioplasty procedure for ACS, reduction of thrombus was very important, because plaque morphology of culprit lesion with the thrombus detected by optical coherence tomography is strongly associated with major adverse cardiac events [26]. In addition, it is important to define the endpoint of PCB angioplasty from the lesion preparation steps to the final PCB dilation step without bailout procedures by DES placement. Thus, for the implementation of PCB angioplasty for ACS cases by the emergent procedures, it is important to gain experience of PCB angioplasty in the cases of CCS by the elective procedures. Finally, preservation of vasomotion of the ischemia related coronary artery after PCB angioplasty was reported [28]. This benefit of PCB angioplasty could also be brought about without DES placement. Coronary vasospasm and hyper-vasocontraction have been known to underlie the pathogenesis of various major cardiac diseases, such as ACS, CCS, MI with non-obstructive coronary arteries (MINOCA), heart failure, malignant ventricular arrhythmia, and sudden death [29]. Collectively, in order to fully elucidate the feasibility of PCB angioplasty in the treatment of ACS, further details with the long-term clinical follow-up after PCB angioplasty including LLE and coronary vasomotion following pre-modulation of the culprit lesions under the guidance of intravascular assessment need to be clarified.

PCB angioplasty has been recognized as the most efficacious method of “stent-less PCI” and “leave-nothing-behind strategy”, as the substitute of DES [1,2,3]. Therefore, PCB-efficacious baseline underlying the present insignificant differences of all of the major cardiovascular outcomes compared to the recent DESs need to be explored. As one of the possibilities, the PCB length of approximately 20 mm (Table 3) was raised. The present approximately 20 mm of PCB length was similar which showed an equivalent (non-inferior) efficacy of PCB angioplasty compared to DES placement in cohorts of DES-ISR [30], and of the three vessel sizes of CCS (small [20], large [22], and the entire vessel size [21]). This was also consistent in the studies estimating the benefits of PCB angioplasty with the intravascular parameters [23,24,26]. Since PCB was used after pre-modification of the culprit plaque, the percentage of heavily calcified lesions in the ACS cohort would not be high (Table 2 and Table S2). The calcified nodule in the culprit of ACS was implicated in as high as 40% of cumulative MACE after PCB angioplasty [26]. The thrombus burden was also associated with cumulative MACE after PCB angioplasty [26]. The necessity of reduction of thrombus burden was mentioned above. Therefore, (1) non-diffuse as in-balloon length of approximately 20 mm, (2) either erosive or ruptured plaques containing small or less thrombus enable reducing by aspiration, and (3) not-heavily calcified lesions enable modifying by a smaller, and shorter semi-compliant or scoring balloons prior to single PCB application would be an optimal indication of PCB in the de novo lesions of ACS cohort. Therefore, intravascular imaging in PCB angioplasty for unstable coronary lesions in patients with ACS have the crucial roles to clarify these points. Thus, since lesions and patients with ACS are heterogeneous with a variety of baseline characteristics, a large-scale prospective study is needed to confirm not only the advantages, but also the disadvantages of PCB angioplasty for patients with ACS by comparing with DES placement in order to explore the optimal indication of the PCB angioplasty in the clinical settings.

The present study is the first meta-analysis showing the PCB angioplasty for patients with ACS, by confirming the impact of PCB angioplasty on a subset of ACS patients with STEMI (Figure 5). The sub-analysis showed the equivalencies of PCB angioplasty for the comparisons of MACE (Figure 5A), CM (Figure 5B), ACM (Figure 5C), and TLR (Figure 5D) with DES placement. A recent meta-analysis showed cumulatively inconclusive to use DCB for STEMI in terms of the significant higher risk for target vessel revascularization (TVR) [31] and numerically higher number of target lesion revascularization (TLR) [32] compared to primary DES placement. As mentioned above, the present subset study included more up-to-date four studies, yielding more than 600 cases in each arm, and showed the statistical safety and efficacy of PCB angioplasty compared with DES placement. However, since the weights of Study No. 9 were very high in the estimations in the four cardiovascular outcomes in the sub-analysis of STEMI (Figure 5A–D), further clinical reports and cumulative meta-analysis including the present meta-analysis would be necessary to support the efficacy of PCB angioplasty for STEMI by comparing with DES placement.

PCB angioplasty has been considered to be able to shorten the duration of dual anti-platelet agents (DAPT) therapy rather than the duration after DES placement [1,2,3]. Therefore, shortening the duration of DAPT has been one of the benefits of PCB angioplasty by decreasing the frequencies of bleeding events compared to DES placement in patients with ACS. The present study showed that bleeding events did not increase after PCB angioplasty compared to DES placement (Figure 3D), although DAPT duration after PCB angioplasty tended to be shorter than after DES placement (Table 3). However, very short duration (one month) DAPT and aspirin-free single anti-platelet agents with only a thienopyridine agent (SAPT) therapy following DES placement for ACS have been reported [33]. Therefore, the difference in the DAPT duration between PCB angioplasty and DES placement, particularly in cases of ACS, needs to be further explored.

There were several limitations in the present systematic review and meta-analysis. First, the total number of ACS cohorts was approximately 2100 cases and the observational interval was within a few years. Thus, cumulative larger scale with long-term observational interval would reconfirm the feasibility of PCB angioplasty, particularly to compare the hard clinical endpoints, mortality, and MI including acute occlusion after PCB angioplasty and stent thrombosis after DES placement. Second, the inclusion and exclusion criteria were varying in the nine studies, and the estimated clinical and angiographic outcomes were not precisely unified (Table 1, Table 2, Table 3, Table 4, Table S2 and Table S3). As mentioned in Discussion, it is very important to take the present interpretation that approximately half of patients with cardiogenic shock (Killip classification of 4) and lesions with heavily calcification which were the consistent predictors of adverse outcome were excluded from the meta-analysis (Table 1, Table 2 and Table S2). Third, although the net cardiovascular outcomes were estimated, the details of procedural processes and methods in both angioplasties, such as lesion preparation, bailout stenting, and the decisions of intravascular findings after pre-modification could not be fully estimated (Table 3, Table 4 and Table S3). Finally, according to the novel statistical methods of meta-analysis, there is a possibility that the present contents were resigned.

## 5. Conclusions

The present systematic review and meta-analysis firstly showed the feasibility of PCB angioplasty for de novo lesions in patients with ACS by the emergent procedures by comparing with DES placement.

## Figures and Tables

**Table 1:** Study design, clinical presentation, population, and definition of major adverse cardiac event of included 9 studies.

Study No	Author/(Study)/Journal	Baseline Adjustment	Types of ACS (n, PCB/DES)	Definition of MACE	Mean Follow-Up Interval
1	Gobic, Am J Med Sci 2017 [9]	RCT	STEMI (38/37)	CM, MI, TLR, and ST	6 months
2	Vos NS (REVELATION), JACC 2019 [6]	RCT	STEMI (58/54)	CM, MI, TLR	9 months
3	Tan, Intern Emerg Med 2021 [10]	Retrospective study (well balanced baselines)	STEMI, and NSTEMI (56/212)	ACM, MI, TLR or TVR	24 months
4	Hao, J Cardioth Surg 2021 [11]	RCT	AMI (STEMI, and NSTEMI) (PCB 24/36, DES 118/94)	CM, MI, TLR	12 months
5	Wang, Circ J 2022 [12]	RCT	STEMI (87/85)	CM, MI, TVR, ST	12 months
6	Mizutani, Int Heart J 2022 [13]	Retrospective study (PSM)	ACS (STEMI, NSTEMI, and UA) (PCB 62/2/27, DES 70/5/19)	CM, MI, TVR, TLR	671 ± 508/626 ± 543 (days)
7	Mangner (BASKET-SMALL2), Circ Cardiovasc Interv 2022 [14]	RCT	ACS (STEMI, NSTEMI, and UA) (whole 15/109/90)	CM, MI, TVR	1 yr, 3 yr
8	Zhang, Clin Appl Thromb Hemost. 2022 [15]	Retrospective study (no significant differences in baseline clinical characteristics, preoperative minimal luminal diameter, and preoperative diameter stenosis)	ACS (STEMI, NSTEMI, and UA) (PCB 7/19/29, DES 12/19/37)	CM, MI, TLR	1 yr
9	Merinopoulos, JACC 2023 [16]	Retrospective study (PSM)	STEMI (439/439)	CM, TLR	1 yr, 3 yr

PCB: paclitaxel-coated balloons, DES: drug-eluting stents, RCT: randomized controlled trial, PSM: propensity score matched analysis, AMI: acute myocardial infarction, STEMI: ST-elevated myocardial infarction, NSTEMI: non-STEMI, ACS: acute coronary syndrome UA: unstable angina, MACE: major adverse cardiac event, CM: cardiac mortality, TLR: target lesion revascularization, ST: stent thrombosis, ACM: all-cause mortality, TVR: target vessel revascularization.

**Table 2:** Patient, angiographic, and lesion characteristics of 9 studies.

	Age (yr)		Male (%)		Diabetes (%)		Smoker (%)		Killip Classification (I/IV) (%)		LAD (%)		Thrombus Containing Lesion or Pre-Aspiration (%)		Calcified Lesion (%)	
Study No	PCB	DES	PCB	DES	PCB	DES	PCB	DES	PCB	DES	PCB	DES	PCB	DES	PCB	DES
1	56.6 ± 13.2	54.3 ± 10.6	71.1	73	5.3	10.8	42.1	56.8	94.0/0	89.2/0	NA	NA	NA	NA	NA	NA
2	57.4 ± 9.2	57.3 ± 8.3	86.7	86.7	13.3	6.7	60.0	50.0	NA/excluded	NA/excluded	31.7	40.0	47.0	50.0	Excluded	Excluded
3	64.96 ± 8.82	62.39 ± 9.91	60.7	65.57	32.14	26.85	51.78	43.51	67.9/5.36	76.4/4.72	33.92	47.16	NA	NA	NA	NA
4	59 ± 11	56 ± 11	75.0	82.0	28	35	28	31	NA/0	NA/0	52	50	None	None	Excluded	Excluded
5	49.20 ± 10.59	49.60 ± 8.82	95.7	91.3	77.2	85.9	77.2	84.8	NA	NA	NA	NA	NA	NA	Excluded	Excluded
6	67.5 ± 12.3	67.9 ± 12.3	81.3	90.2	36.3	36.3	NA	NA	NA/18.7	NA/18.7	53.8	50.5	41.8	38.5	4.4	2.2
7	68.3 ± 10.7	68.3 ± 10.7	67.8	67.8	31.4	31.4	48.6	48.6	NA	NA	36.5	36.5	NA	NA	NA	NA
8	56.40 ± 10.24	56.04 ± 9.36	67.3	80.9	38.2	22.1	34.5	32.4	NA	NA	50.9	61.8	NA	NA	Excluded	Excluded
9	66 ± 13	66 ± 11	73.0	74.0	14.0	12.0	58.0	67.0	NA/excluded	NA/excluded	40.0	43.0	15.0	13.0	14.0	13.0

LAD: left ascending artery. In Study No.6, the percentages of calcified lesions decreased from crude cohort to adjusted cohort. NA: Not Available.

**Table 3:** Characteristics of final device, and dual anti-platelet therapy (DAPT).

Study No	Used PCB	Used DES	Bailout Stent Implantation (%)	Balloon Diameter of PCB and DES (mm)	Total Balloon Length of PCB and DES (mm)	DAPT Regimen (PCB/DES)	DAPT Duration (PCB/DES)
1	SeQuent Please DCBs	Cobalt-chromium SES (Biomime, MerilLifeSciences, Vapi, India)	4.0	80–100% that of the reference diameter of the culprit coronary artery	culprit lesion itself and 2 mm from both sides of the lesion were covered	Dual antiplatelet therapy according to current European Society of Cardiology guidelines	1 year
2	Pantera Lux	Orsiro, or Xience	0	NA	NA	ASA + clopidogrel or ticagrelor	all of 9 months
3	SeQuent Please DCBs	zotarolimus-eluting stent or rapamycin-eluting stent	NA	2.61 ± 0.19/2.63 ± 0.16	22.41 ± 7.10/27.62 ± 15.82	ASA + clopidogrel/ASA + clopidogrel or ticagrelor	3 months/12 months
4	Yinyi (Liaoning) Biotech BingoDrug Coated Balloon	NA	NA	The ratio of the diameter of PCB to the diameter of the normal segment of the target blood vessel is 1.1:1	the ratio of the length of the implanted stent to the diseased segment is 1.3:1	aspirin + clopidogrel	6 months/12 months
5	An ultrasound-controlled paclitaxel releasing balloon	CordimaxTM stents	NA	3.19 ± 0.57/3.34 ± 0.49	PCBs are available in lengths from 15 to 35 mm	ASA and clopidogrel	3 months/12 months
6	SeQuent Please DCBs	7 recent DESs	6.14	3.02 ± 0.547/3.04 ± 0.551	20.9 ± 6.23/20.5 ± 6.17	ASA + clopidogrel or prasugrel	338 ± 328/537 ± 567 (days)
7	Paclitaxel-coated SeQuent Please or SeQuent Please Neo balloon	Everolimus-eluting Xience stent or paclitaxel-eluting Taxus Element stent	NA	2.6 ± 0.3/2.6 ± 0.3	19.9 ± 4.4/19.0 ± 6.0	ASA and either clopidogrel, prasugrel, or ticagrelor	4 weeks/12 months
8	Paclitaxel/iohexol matrix coating on the Bingo Drug-Coated Balloon	NA	NA	3.09 ± 0.58/3.18 ± 0.46	21.60 ± 6.92/23.71 ± 7.66	aspirin + ticagrelor, twice a day	NA
9	Paclitaxel drug coated balloon	second-generation DES	excluded (5.31%)	NA	NA	NA	348 ± 78/346 ± 76 (days)

DAPT: dual anti-platelet therapy, ASA: acetylsalicylic acid (aspirin).

**Table 4:** Angiographic parameters in PCB angioplasty and DES placement.

Baseline Reference Vessel Diameter (mm)		Minimal Luminal Diameter after PCI (mm)		Percent Diameter Stenosis after PCI		Late Luminal Loss (mm)			
PCB	DES	PCB	DES	PCB	DES	PCB	DES	*p*-Values	Late Lumen Enlargement (%, n/N)
2.61 ± 0.49 (n = 32)	3.04 ± 0.46 (n = 31)	2.47 ± 0.50 (n = 31)	2.68 ± 0.61 (n = 32)	NA	NA	(−0.09 ± 0.09) (n = 31)	0.10 ± 019 (n = 32)	<0.05	NA
3.28 ± 0.52 (n = 42)	3.20 ± 0.48 (n = 38) (*p* = 0.35)	2.64 ± 0.42 (n = 42)	2.88 ± 0.41 (n = 42)	NA	NA	0.05 (n = 42)	0.00 (n = 42)	0.51	NA
2.64 ± 0.17 (n = 56)	2.65 ± 0.14 (n = 212) (*p* = 0.625)	NA	NA	NA	NA	0.14 ± 0.13 (n = 20)	0.19 ± 012 (n = 58)	0.442	NA
2.5–4.0	2.5–4.0	2.85 ± 0.28 (n = 31)	3.17 ± 0.36 (n = 36) (*p* < 0.05)	NA	NA	(−0.12 ± 0.46) mm (n = 38)	0.14 ± 0.37 mm (n = 42)	<0.05	NA
3.31 ± 0.56 (n = 92)	3.43 ± 0.48 (n = 92) (*p* = 0.120)	2.71 ± 0.53 (n = 92)	2.92 ± 0.44 (n = 92) (*p* = 0.001)	18.12 ± 5.91 (n = 92)	14.61 ± 5.01 (n = 92) (*p* < 0.01)	0.24 ± 0.39 (n = 77)	0.31 ± 0.38 (n = 74)	0.266	NA
2.44 ± 0.560 (n = 91)	2.83 ± 0.617 (n = 91) (*p* < 0.001)	1.97 ± 0.548 (n = 91)	2.47 ± 0.565 (n = 91) (*p* < 0.001)	19.6 ± 10.6 (n = 91)	12.4 ± 5.98 (n = 91) (*p* < 0.001)	0.034 ± 0.578 (n = 54)	0.473 ± 0.764 (n = 59)	0.002	48.1 (26/54)
NA	NA	NA	NA	NA	NA	NA	NA	NA	NA
3.01 ± 0.64 (n = 55)	3.06 ± 0.41 (n = 68) (*p* = 0.571)	2.23 ± 0.56 (n = 55)	2.60 ± 0.38 (n = 68) (*p* < 0.01)	24.95 ± 0.56 (n = 55)	2.60 ± 0.38 (n = 68) (*p* < 0.01)	0.02 (n = 55)	0.24 (n = 68)	0.03	56.4 (31/55)
3.50 (3.00–3.50)	3.50 (3.00–4.00) (*p* < 0.001)	NA	NA	NA	NA	NA	NA	NA	NA

## Data Availability

Not applicable.

## Supplementary Materials

The following supporting information can be downloaded at: https://www.mdpi.com/article/10.3390/jcm13051481/s1, Table S1 PRISMA 2020 checklist. Table S2 Inclusion and exclusion criteria of 9 studies. Abbreviations were the same with Table 1. DCB: drug-coated balloons, TIMI grade flow: thrombolysis in MI, DAPT: dual antiplatelet therapy, PCI: percutaneous coronary intervention, ISR: in-stent restenosis. Table S3 Lesion preparation and treatment of dissection during PCB angioplasty. Abbreviations were the same with other Tables.
