# Supplementary material for: Cardiovascular Outcomes after Paclitaxel-Coated Balloon Angioplasty versus Drug-Eluting Stent Placement for Acute Coronary Syndrome: A Systematic Review and Meta-Analysis
Source: J Clin Med. 2024 Mar 4;13(5):1481. doi: 10.3390/jcm13051481 (PMC10934935; doi:10.3390/jcm13051481)
Supplement: Supplementary file 1 [file jcm-13-01481-s001.zip › jcm-2848937-supplementary.pdf]

Table S1. PRISMA 2020 checklist according to the The PRISMA 2020 statement [17]. The number of page and Tables were corresponded to the text and supplement.

Section and Topic	Item #	Checklist item	Location where item is reported
**TITLE**			
Title	1	Identify the report as a systematic review.	Page 1
**ABSTRACT**			
Abstract	2	See the PRISMA 2020 for Abstracts checklist.	Page 1
**INTRODUCTION**			
Rationale	3	Describe the rationale for the review in the context of existing knowledge.	Pages 1-2
Objectives	4	Provide an explicit statement of the objective(s) or question(s) the review addresses.	Pages 1-2
**METHODS**			
Eligibility criteria	5	Specify the inclusion and exclusion criteria for the review and how studies were grouped for the syntheses.	Page 2
Information sources	6	Specify all databases, registers, websites, organisations, reference lists and other sources searched or consulted to identify studies. Specify the date when each source was last searched or consulted.	Page 2
Search strategy	7	Present the full search strategies for all databases, registers and websites, including any filters and limits used.	Pages 2-3 (Figure 1)
Selection process	8	Specify the methods used to decide whether a study met the inclusion criteria of the review, including how many reviewers screened each record and each report retrieved, whether they worked independently, and if applicable, details of automation tools used in the process.	Page 2
Data	9	Specify the methods used to collect data from reports, including how many	Pages 2-3

Section and Topic	Item #	Checklist item	Location where item is reported
collection process		reviewers collected data from each report, whether they worked independently, any processes for obtaining or confirming data from study investigators, and if applicable, details of automation tools used in the process.	
Data items	10a	List and define all outcomes for which data were sought. Specify whether all results that were compatible with each outcome domain in each study were sought (e.g. for all measures, time points, analyses), and if not, the methods used to decide which results to collect.	Pages 2-3, Table S2
	10b	List and define all other variables for which data were sought (e.g. participant and intervention characteristics, funding sources). Describe any assumptions made about any missing or unclear information.	Pages 2-3, and Tables S2-S3AB
Study risk of bias assessment	11	Specify the methods used to assess risk of bias in the included studies, including details of the tool(s) used, how many reviewers assessed each study and whether they worked independently, and if applicable, details of automation tools used in the process.	Page 2
Effect measures	12	Specify for each outcome the effect measure(s) (e.g. risk ratio, mean difference) used in the synthesis or presentation of results.	Figures 2 and 3, and Table S5
Synthesis methods	13a	Describe the processes used to decide which studies were eligible for each synthesis (e.g. tabulating the study intervention characteristics and comparing against the planned groups for each synthesis (item #5)).	Pages 3-4
	13b	Describe any methods required to prepare the data for presentation or synthesis, such as handling of missing summary statistics, or data conversions.	NA
	13c	Describe any methods used to tabulate or visually display results of individual	Figures 2 and 3, and Table S5

Section and Topic	Item #	Checklist item	Location where item is reported
		studies and syntheses.	
	13d	Describe any methods used to synthesize results and provide a rationale for the choice(s). If meta-analysis was performed, describe the model(s), method(s) to identify the presence and extent of statistical heterogeneity, and software package(s) used.	Pages 3-4
	13e	Describe any methods used to explore possible causes of heterogeneity among study results (e.g. subgroup analysis, meta-regression).	Figure 2B
	13f	Describe any sensitivity analyses conducted to assess robustness of the synthesized results.	NA
Reporting bias assessment	14	Describe any methods used to assess risk of bias due to missing results in a synthesis (arising from reporting biases).	NA
Certainty assessment	15	Describe any methods used to assess certainty (or confidence) in the body of evidence for an outcome.	Figure 2B
**RESULTS**			
Study selection	16a	Describe the results of the search and selection process, from the number of records identified in the search to the number of studies included in the review, ideally using a flow diagram.	Figure 1
	16b	Cite studies that might appear to meet the inclusion criteria, but which were excluded, and explain why they were excluded.	Figure 1
Study characteristics	17	Cite each included study and present its characteristics.	Page 4, and Table S2

Section and Topic	Item #	Checklist item	Location where item is reported
Risk of bias in studies	18	Present assessments of risk of bias for each included study.	Page 1,and Table S2
Results of individual studies	19	For all outcomes, present, for each study: (a) summary statistics for each group (where appropriate) and (b) an effect estimate and its precision (e.g. confidence/credible interval), ideally using structured tables or plots.	Figures 2 and 3
Results of syntheses	20a	For each synthesis, briefly summarise the characteristics and risk of bias among contributing studies.	NA
	20b	Present results of all statistical syntheses conducted. If meta-analysis was done, present for each the summary estimate and its precision (e.g. confidence/credible interval) and measures of statistical heterogeneity. If comparing groups, describe the direction of the effect.	Figures 2 and 3
	20c	Present results of all investigations of possible causes of heterogeneity among study results.	Figure 2 and 3
	20d	Present results of all sensitivity analyses conducted to assess the robustness of the synthesized results.	NA
Reporting biases	21	Present assessments of risk of bias due to missing results (arising from reporting biases) for each synthesis assessed.	NA
Certainty of evidence	22	Present assessments of certainty (or confidence) in the body of evidence for each outcome assessed.	Figure 2B
**DISCUSSION**			
Discussion	23a	Provide a general interpretation of the results in the context of other evidence.	Pages 8-10
	23b	Discuss any limitations of the evidence included in the review.	Pages 9-10

Section and Topic	Item #	Checklist item	Location where item is reported
	23c	Discuss any limitations of the review processes used.	Pages 9-10
	23d	Discuss implications of the results for practice, policy, and future research.	Pages 8-10
**OTHER INFORMATION**			
Registration and protocol	24a	Provide registration information for the review, including register name and registration number, or state that the review was not registered.	Page 2
	24b	Indicate where the review protocol can be accessed, or state that a protocol was not prepared.	NA
	24c	Describe and explain any amendments to information provided at registration or in the protocol.	NA
Support	25	Describe sources of financial or non-financial support for the review, and the role of the funders or sponsors in the review.	Supple
Competing interests	26	Declare any competing interests of review authors.	Supple
Availability of data, code and other materials	27	Report which of the following are publicly available and where they can be found: template data collection forms; data extracted from included studies; data used for all analyses; analytic code; any other materials used in the review.	Not repost (both of the first and corresponding authors have)

Table S2. Inclusion and exclusion criteria of 9 studies. Abbreviations were the same with Table 1. DCB: drug-coated balloons, TIMI grade flow: thrombolysis in MI, DAPT: dual antiplatelet therapy, PCI: percutaneous coronary intervention, ISR: in-stent restenosis.

Study No	Author/(Study)/Journal	Study design including enrolled interval and population	Exclusion criteria
1	Gobic, Am J Med Sci 2017	Seventy-five patients with STEMI during from March2014 to January2015 with de novo lesions in vessel diameter of 2.5-4.0 mm were randomized into DES and PCB groups of 37 and 38 patients	11 criteria
2	Vos NS (REVELATION), JACC 2019	A prospective, randomized, single-center REVELATION trial, comparing PCB with DES in 120 patients presenting with STEMI. Patients with a new, nonseverely calcified culprit lesion in a native coronary artery and a residual stenosis of <50% after pre-dilatation were randomized to treatment with a PCB or DES.	Patients with a history of MI, stent implantation <1 month, contraindications for DAPT or anticoagulation therapy, and cardiogenic shock or intubation before randomization
3	Tan, Intern Emerg Med 2021	Between March 2016 and March 2018, patients of AMI (STEMI and NSTEMI) with de novo small coronary artery (reference diameter 2.0-2.8 mm) and received percutaneous coronary intervention (PCI) were enrolled. 268 patients were divided into DEB group (PCI with further DEB, n = 56) and drug-eluting stent (DES) group (PCI with further DES, n = 212).	unprotected left main lesion, ISR, cardiogenic shock, chronic total occlusions, heavily calcified
4	Hao, J Cardioth Surg 2021	STEMI patients who were hospitalized in the hospital from January 2018 to December 2019 and received emergency PCI treatment. Inclusion criteria: 18-80 years old; Patients diagnosed with STEMI and receiving emergency PCI; The duration from onset to vascular opening <= 12 hours; New coronary artery disease (occlusion or severe stenosis). The reference vessel diameter is 2.5–4.0 mm, and there is no severe calcification.	7 criteria including cardiogenic shock or cardiac arrest;

5	Wang, Circ J 2022	184 pretreated STEMI patients without C-F dissection and/or TIMI grade flow < 3 were randomized into PCB (n=92) and DES (n=92) groups with a 1:1 allocation during from 2017 October to 2019 August.	>3 coronary diseases, left main coronary artery lesions, and severely distorted or calcified or angulated vessels
6	Mizutani, Int Heart J 2022	A total of 309 consecutive de novo native coronary lesions in patients with ACS who were successfully (TIMI-grade flow of 2 or 3) treated by emergent procedures using either a PCB (n = 107) or a DES between January 2016 and December 2019. A propensity score-matched analysis was used to adjust the 36 baseline variables.	all comer continuous cases
7	Mangner (BASKET-SMALL2), Circ Cardiovasc Interv 2022	BASKET-SMALL 2 randomized 758 patients with small vessel coronary artery disease to PCB or DES treatment from 2012 to 2020.	a concomitant PCI of lesions ≥3 mm in diameter in the same epicardial coronary artery, PCI of in-stent restenosis, life expectancy of <12 months, pregnancy, enrollment in another randomized trial, or inability to give informed consent.
8	Zhang, Clin Appl Thromb Hemost. 2022	123 patients with IVUS confirmed vulnerable plaques were retrospectively analyzed and diagnosed with ACS and given PCI by either PCB (n =55) or DES (n =68) in Cardiology Department from December 2020 to July 2022. Inclusion criteria: (1) 18-85 years of age; (2) meeting the diagnostic criteria of the European Heart Association for ACS (3) having definite culprit vessel; and (4) gray-scale IVUS showing PB >= 70% and minimal luminal area (MLA) <= 4.0 mm2.	1) chronic total occlusion; (2) heavily calcified lesions requiring rotational atherectomy; (3) patients with thrombus aspiration; (4) severe valvular insufficiency or valvular stenosis; (5) definite contraindications to antiplatelet drugs or anticoagulants; (6) dissection repair remedial stents after DCB implantation; (7) severe renal insufficiency (GFR< 30 ml/min) or severe liver insufficiency; and (8) patients who declined DCB and DES implantation.
9	Merinopoulos, JACC 2023	The first 1,139 STEMI due to de novo disease were dedicated an investigator-initiated, single-center, retrospective, propensity-matched cohort studytreated with PCBs (n=452) and DES (n=687) between from January 1, 2016 to November 15, 2019.	cardiac arrest, intubation, or cardiogenic shock, as their outcomes are determined mainly bythe severity of the clinical presentation rather than the treatment strategy, bailout stenting

Table S3. Lesion preparation and treatment of dissection during PCB angioplast. Abbreviations were the same with other Tables.

Study No	Lesion preparation for culprit lesion	Thrombectomy	Treatment of dissection during PCB angioplasty
1	After the guiding catheter insertion, guidewire placement and thrombus aspiration, balloon dilation was performed with a balloon catheter of upto 75% of the culprit coronary artery diameter. The DCB was deployed in patients with residual diameter stenosis less than 30% and without type C-F dissection.	NA	Patients with persistent residual stenosis or occurrence of clinically significant dissection which led tobail-out BMS implantation.
2	residual stenosis was #50% (by visual assessment) after thrombus aspiration (in case of visible thrombus) for homogeneous delivery of paclitaxel and mandatory pre-dilatation.	Thrombus aspiration was conducted in case of visible thrombus. Although in large trials the use of routine thrombus aspiration did not affect mortality, this may be more valuable in a DCB strategy because of the importance of optimal lesion preparation before DCB usage and the need for homogeneous delivery of paclitaxel.	Bailout stenting with a bare-metal stent was advised only in case of residual stenosis of the treated lesion >50% (by visual assessment) after balloon dilatations with sufficiently large balloons, or coronary dissection greater than or equal to type C leading to (threatening) vessel closure
3	Predilation of the target lesion was performed in both groups using 1.25–2.5-mm conventional balloon	NA	operator's discretion
4	the thrombus was aspirated. In the Yinyi (Liaoning) Biotech Bingo Drug Coated Balloon group, a semi-compliant balloon was used for expansion. If the dilation reaches the residual diameter stenosis ≤ 30%, and there is no CF type dissection, the dilation is considered to meet the criteria, indicating that the patient can receive DES treatment.	After the guide wire were placed, the thrombus was aspirated.	If CF-type anatomy occurs after expansion, the patient will be treated with a drug-eluting stent
5	successful predilatation (residual stenosis ≤30% and no limited flow dissection or thrombus)	Thrombolysis and thrombus aspiration are necessary for patients with heavy thrombotic load	type C-F were excluded

6	semi-compliant balloon, scoring balloon, thrombus aspiration	thrombus reduction by thrombectomy before PCB or DES implantation	bailout stenting with DES
7	Successful predilatation of the lesion with the absence of higher grade dissections (National Heart, Lung, and Blood Institute grade C to F), decreased blood flow (thrombolysis in myocardial infarction score ≤2), or residual stenosis >30% was obligatory for randomization	NA	DES was recommended
8	For TIMI grade < 3 culprit vessels, an appropriate compliant balloon, semi-compliant balloon, cutting balloon, or spinous balloon was used to dilate the lesion and reduce culprit stenosis was used to pre-dilate the culprit vessel lesions to restore TIMI grade 3 flow.	excluded	For DCB implantation, if the degree of vascular stenosis was ≤ 30%, and there was no dissection or only type A or B dissection after dilation, a size-matched DCB was chosen based on IVUS imaging and placed in the lesion sustained release before balloon withdrawal.
9	NA	NA	NA
